# Supplementary material for: Acetylation is required for full activation of the NLRP3 inflammasome
Source: Nat Commun. 2023 Dec 18;14:8396. doi: 10.1038/s41467-023-44203-0 (PMC10728138; doi:10.1038/s41467-023-44203-0)
Supplement: Supplementary file 1 — Supplementary Information [file 41467_2023_44203_MOESM1_ESM.pdf]

Supplementary Information

Acetylation is required for full activation of the NLRP3 inflammasome

Yening Zhang[1,2], Ling Luo[1], Xueming Xu[1], Jianfeng Wu[3], Fupeng Wang[1], Yanyan Lu[4], Ningjie Zhang[5], Yingying Ding[6], Ben Lu[1,2]* Kai Zhao[1,2]*

[1]Department of Hematology and Critical Care Medicine, the Third Xiangya Hospital, Central South University, Changsha, Hunan Province, 410000 P. R. China
[2]Key Laboratory of Sepsis Translational Medicine of Hunan, Central South University, Changsha, Hunan Province, 410000 P. R. China
[3]State Key Laboratory of Cellular Stress Biology Innovation Center for Cell Signaling Network, School of Life Sciences, Xiamen University, Xiamen, Fujian Province, 361005 P. R. China
[4]Department of Hematology, The Second Xiangya Hospital, Central South University, Changsha, Hunan Province, 410000 P. R. China
[5]Department of Blood Transfusion, The Second Xiangya Hospital, Central South University, Changsha, Hunan Province, 410000 P. R. China
[6]Department of Pathogen Biology, NavaMedical University, Shanghai, 200082 P. R. China,

* Corresponding authors: xybenlu@csu.edu.cn; kaizhao@csu.edu.cn

Supplementary Figures and Figure legends

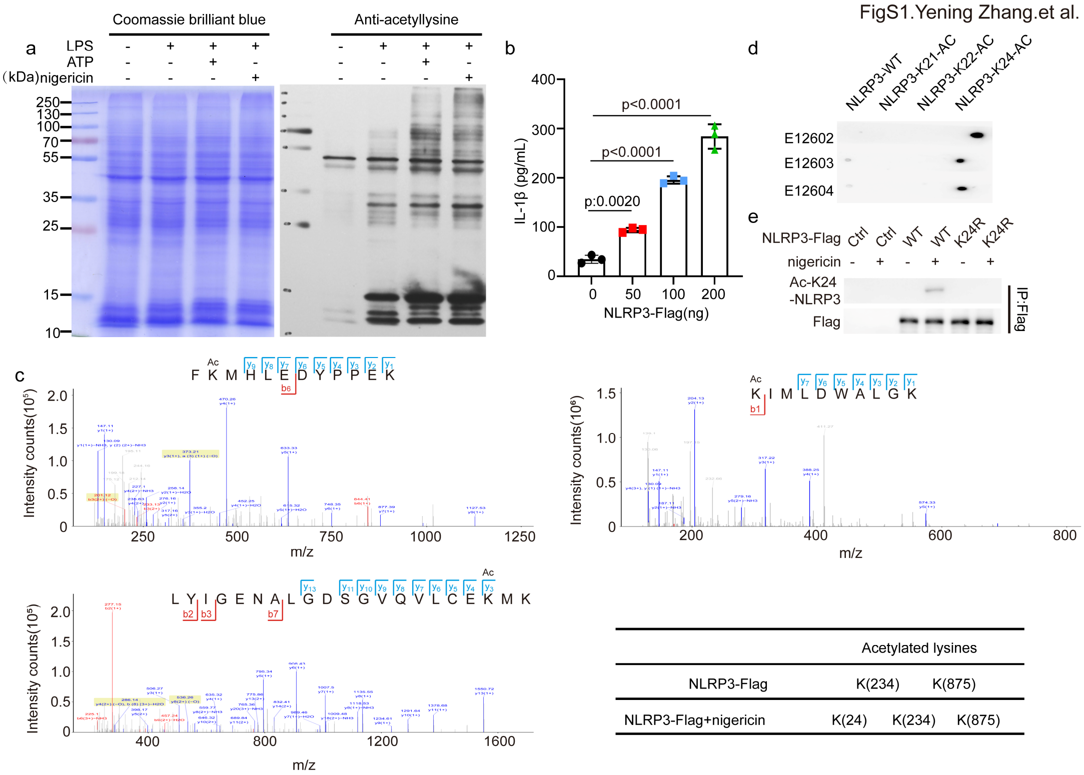

	Acetylated lysines		
NLRP3-Flag	K(234)	K(875)	
NLRP3-Flag+nigericin	K(24)	K(234)	K(875)

Supplementary Figure 1. Identification of NLRP3 K24 acetylation.

a Coomassie bright blue staining and Immunoblot analysis of total acetylation level from lysates of peritoneal macrophages treated with LPS (100 ng/mL, 3 h) along or with ATP (5 mM, 1 h), nigericin (10 μM, 1 h). **b** ELISA analysis of IL-1β in supernatants of HEK293T cells reconstituted with NLRP3 inflammasome and stimulated with nigericin (10 μM,1 h). **c** NLRP3 acetylation sites identified with mass spectrometry. **d-e** Immunoblot analysis of sensitivity and specificity of generated acetyl-lys^{24}-NLRP3 antibody. **d** acetyl-lys^{24}-NLRP3 antibody were tested on the peptides. **e** acetyl-lys^{24}-NLRP3 antibody were tested on HEK293T cells transfected with different NLRP3 plasmid treated with nigericin or not. Results are represented as mean± SD and typical photographs are representative of three biological independent experiments with similar results. Statistical analyses were carried out via one-way ANOVA with Dunnett’s test for **b**. Source data are provided as a Source Data file.

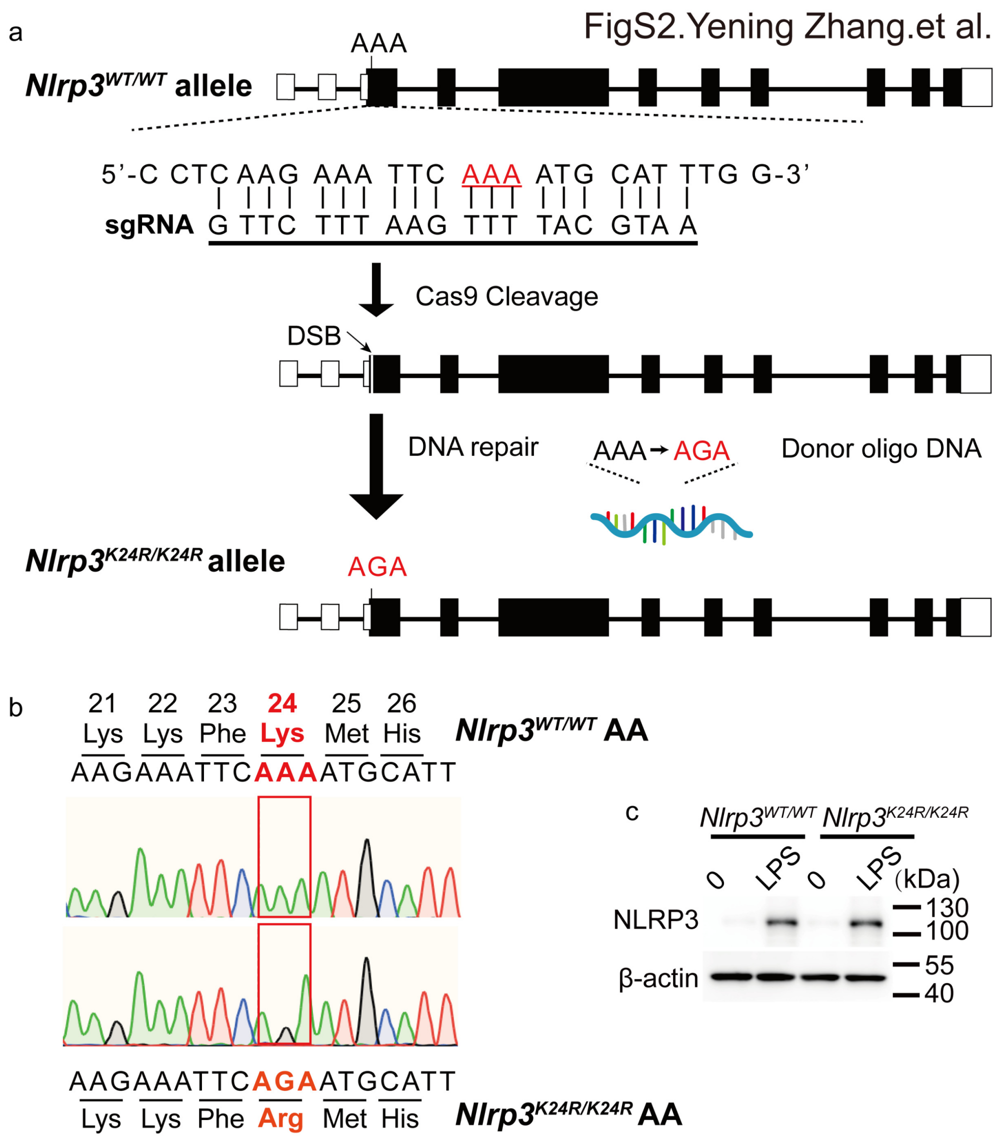

Supplementary Figure 2. Generation of *Nlrp3*$^{K24R/K24R}$ knock-in mice

a Schematic diagram of the strategy for generating *Nlrp3*$^{K24R/K24R}$ knock-in mice. *Nlrp3*$^{K24R/K24R}$ knock-in C57BL/6 mice were generated by the CRISPR-Cas9 technology. The guide RNA (gRNA) binding site on the homologous DNA is indicated, mutated site is underlined and marked in red. **b** Genotyping of NLRP3-mutated mice. NLRP3 K24R mutation was validated by DNA sequencing. **c** Immunoblot analysis of NLRP3 expression of BMDMs from *Nlrp3*$^{WT/WT}$and *Nlrp3*$^{K24R/K24R}$ mice treated with LPS (100 ng/mL,3 h) or not. Typical photographs are representative of three biological independent experiments with similar results.

FigS3.Yening Zhang.et al.

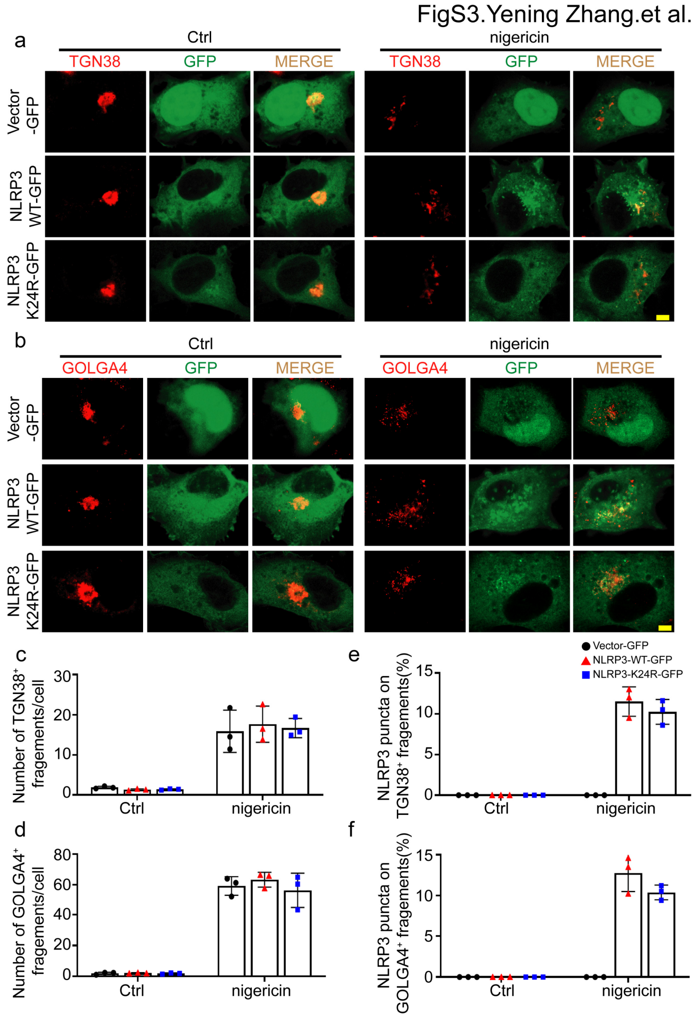

Supplementary Figure 3. K24 acetylation of NLRP3 has no effect on the formation of dTGN.

a-e COS-7 cells were transfected with Vector-GFP or NLRP3(WT/K24R)-GFP plasmids and stimulated with nigericin or not (10 μM, 1h). n=3 biologically independent experiments. **a, b** Representative fluorescent microscopy images of intracellular Co-localization of NLRP3 with the TGN markers GOLGA4 or TGN38. Scale bar = 2 μm. **c, d** Statistical analysis of dispersion of TGN. **e, f** Co-localization analysis of dTGN and NLRP3-GFP puncta. Statistical analyses were carried out via two-way ANOVA with the Bonferroni test for **c-f.** Source data are provided as a Source Data file.

FigS4.Yening Zhang.et al.

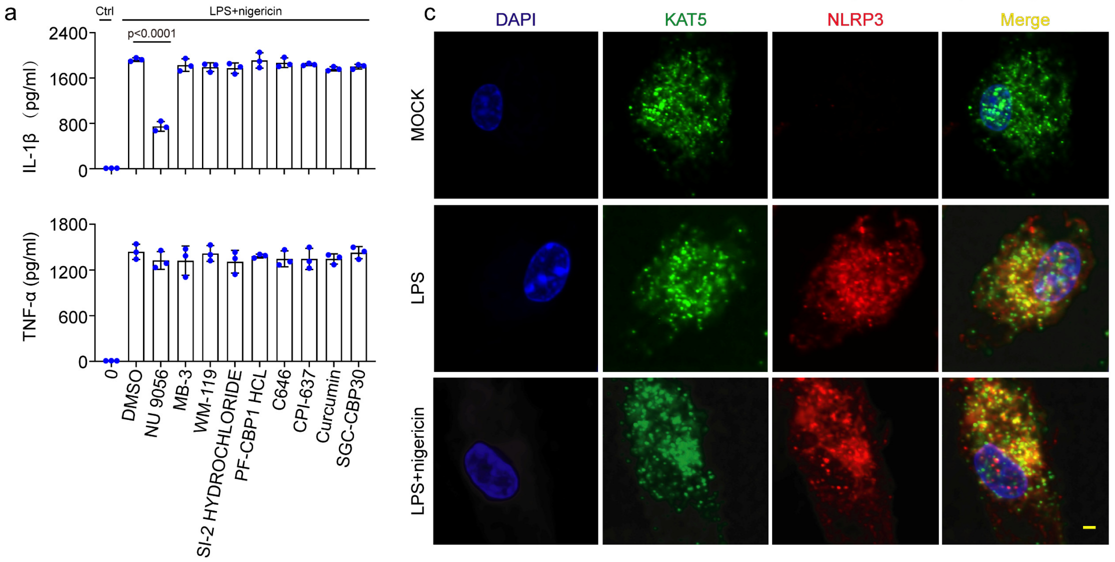

	Target
NU 9056	KAT5
MB-3	KAT2
WM-119	KAT6A
SI-2 HYDROCHLORIDE	KAT13B
PF-CBP1 HCL	KAT3A
C646	KAT3B
CPI-637	KAT3B
Curcumin	KAT3B
SGC-CBP30	KAT3A/3B

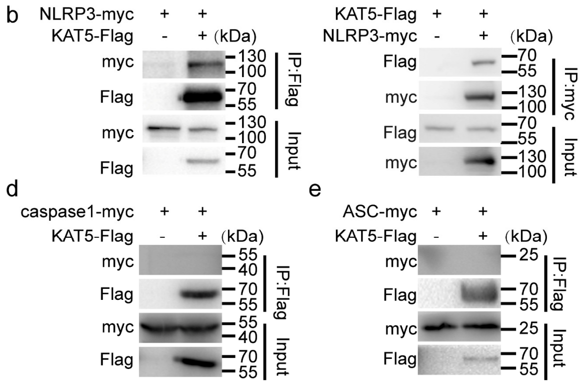

Supplementary Figure 4. KAT5 is involved in NLRP3 inflammasome activation.

a ELISA of IL-1β and TNF-α in supernatants from LPS-primed (100 ng/mL, 3h) mouse peritoneal macrophages treated with 1μM various acetyltransferase inhibitors and then stimulated with nigericin (10 μM, 1 h). n=3 biologically independent experiments. **b** Co-IP analysis of interaction between KAT5 with NLRP3 from HEK293T cells transfected with myc-tagged NLRP3 and Flag-tagged KAT5. **c** Representative fluorescent microscopy images of intracellular Co-localization of KAT5 and NLRP3 in peritoneal macrophages treated with LPS (100 ng/mL, 3 h) along or with nigericin (10 μM, 1 h). Scale bar = 2μm. **d, e** Co-IP analysis of interaction between KAT5 with caspase-1 or ASC from HEK293T cells transfected with myc-tagged caspase-1 or ASC with Flag-tagged KAT5. Results are represented as mean± SD and typical photographs are representative of three biological independent experiments with similar results. Statistical analyses were carried out via one-way ANOVA with Dunnett's test for **a**. Source data are provided as a Source Data file.

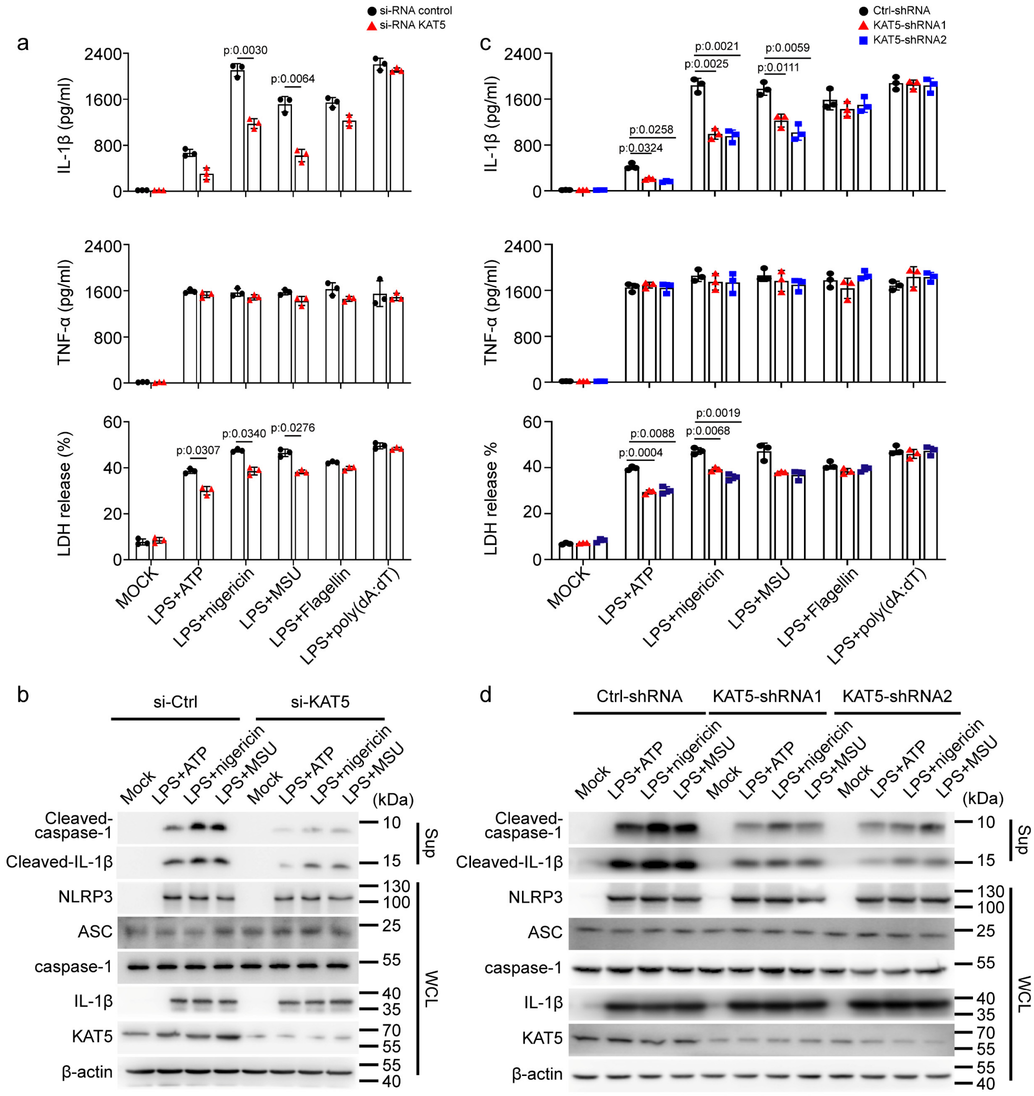

Supplementary Figure 5. KAT5 promotes NLRP3 inflammasome activation.

a, b Mouse peritoneal macrophages transfected with siRNA control or KAT5 were treated with LPS (100 ng/mL, 3 h) along or with ATP (5 mM, 1 h), nigericin (10 μM, 1 h), MSU (200 μg/mL, 6 h), Flagellin transfection (2 μg/mL, 1 h) or poly (dA:dT) transfection (1 μg/mL, 16 h). n=3 biologically independent experiments. **a** ELISA analysis of IL-1β, TNF-α and release of LDH in supernatants. **b** Cell Lysates and supernatant were subjected to western blot analysis. casp-1(caspase-1). **c, d** iBMDMs stably expressing shRNAs targeting KAT5(shRNA-1and shRNA-2) were treated with LPS (1μg/mL, 6 h) along or with ATP (10 mM, 1 h), nigericin (20 μM, 1 h), MSU (400 μg/mL, 6 h), Flagellin transfection (4 μg/mL, 1h) or poly (dA:dT) transfection (2 μg/mL, 16 h). **c** ELISA analysis of IL-1β, TNF-α and release of LDH in supernatants. **d** Cell

Lysates and supernatant were subjected to western blot analysis. Results are represented as mean± SD and typical photographs are representative of three biological independent experiments with similar results. Statistical analyses were carried out via two-way ANOVA with the Bonferroni test for **a, c**. Source data are provided as a Source Data file.

FigS6.Yening Zhang.et al.

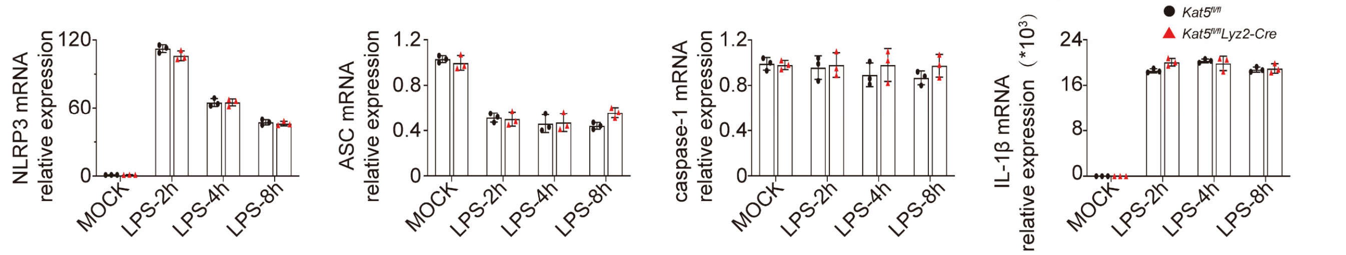

Supplementary Figure 6. KAT5 have no effects on the mRNA expression of NLRP3 inflammasome components.

Relative NLRP3, ASC, casepase-1, IL-1β mRNA expression of *Kat5*$^{fl/fl}$ or *Kat5*$^{fl/fl}$*Lyz2-Cre* BMDMs challenged with LPS (100 ng/mL) for indicated time (0, 2, 4 or 8 h). n=3 biologically independent experiments. Results are represented as mean± SD. Statistical analyses were carried out via two-way ANOVA with the Bonferroni test. Source data are provided as a Source Data file.

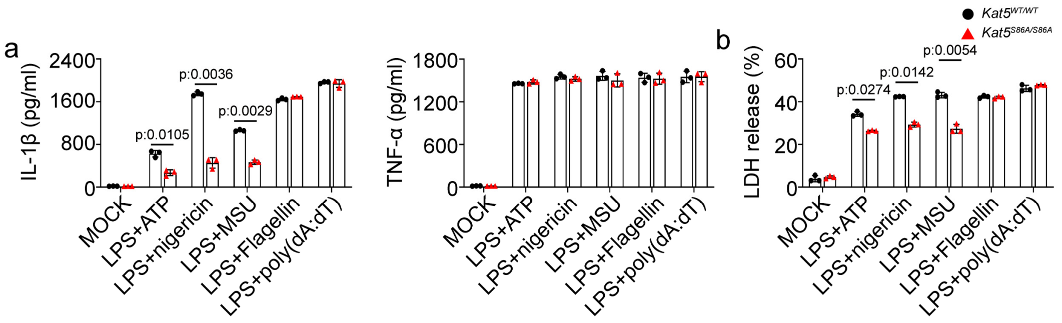

Supplementary Figure 7. ***Kat5*** $^{\boldsymbol{S86A/S86A}}$ **inhibits NLRP3 inflammasome activation**

a, b *Kat5* $^{WT/WT}$ or *Kat5* $^{S86A/S86A}$ BMDMs were treated with LPS (100 ng/mL, 3 h) along or with ATP (5 mM, 1 h), nigericin (10 μM, 1 h), MSU (200 μg/mL, 6 h), Flagellin transfection (2 μg/mL, 1 h) or poly (dA:dT) transfection (1 μg/mL, 16 h). n=3 biologically independent experiments. **a** ELISA of IL-1β, TNF-α in supernatants. **b** release of LDH in supernatants. Results are represented as mean ± SD. Statistical analyses were carried out via two-way ANOVA with the Bonferroni test for **a, b**. Source data are provided as a Source Data file.

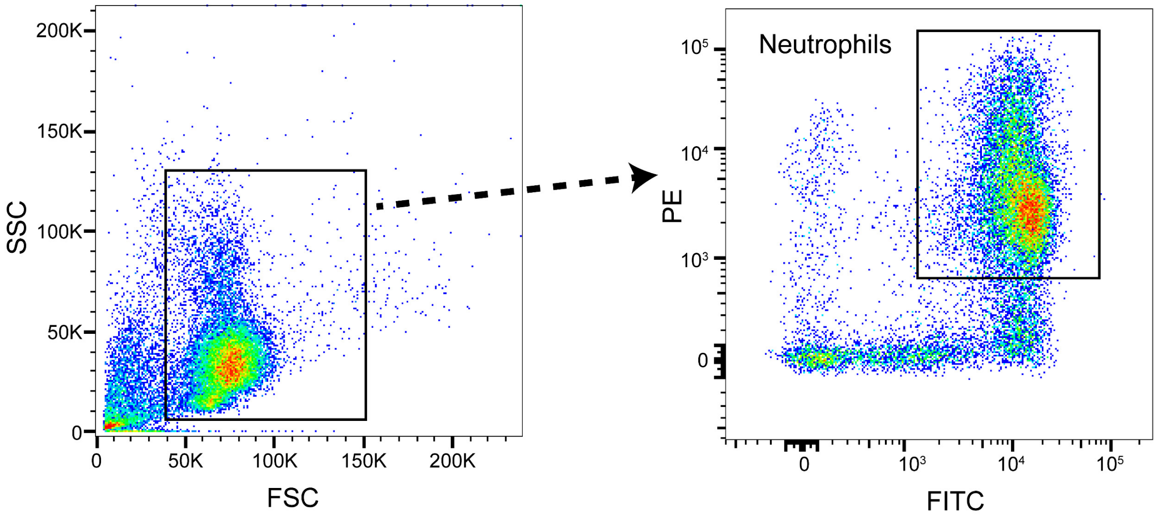

Supplementary Figure 8. Gate strategies of neutrophils in peritoneal lavage fluid

Peritoneal lavage cells were assessed by flow cytometry. The Gated neutrophils are FITC+ PE+.

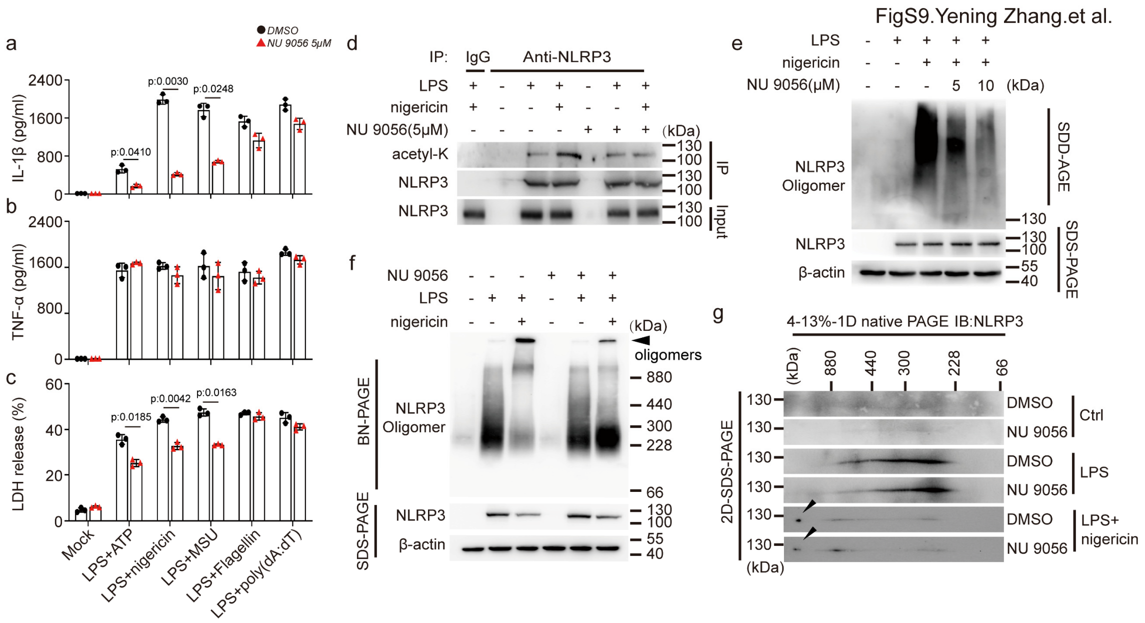

Supplementary Figure 9. NU9056 specifically blocks NLRP3 inflammasome activation *ex vivo*.

a-f LPS-primed (100 ng/mL, 3 h) mouse peritoneal macrophages treated with or without NU 9056 for 30 min followed with LPS (100 ng/mL, 3 h) along or with ATP (5 mM, 1 h), nigericin (10 μM, 1 h), MSU (200 μg/mL, 6 h), Flagellin transfection (2 μg/mL, 1 h) or poly (dA:dT) transfection (1 μg/mL, 16 h). n=3 biologically independent experiments. **a, b** ELISA of IL-1β, TNF-α in supernatants. **c** release of LDH in supernatants. **d** Immunoblot analysis of acetylation level of NLRP3. **e-g** Immunoblot analysis of NLRP3 oligomerization and NLRP3 expression by SDD-AGE, BN-PAGE and 2D-SDS-PAGE analysis. Results are represented as mean ± SD and typical photographs are representative of three biological independent experiments with similar results. Statistical analyses were carried out via two-way ANOVA with the Bonferroni test for **a-c**. Source data are provided as a Source Data file.

Supplementary Table1

REAGENT or RESOURCE	SOURCE	IDENTIFIER
Antibodies		
Anti-caspase-1 antibody	abcam	ab179515
Anti-IL-1β antibody	RD systems	AF-401-NA; RRID: AB_416684
Anti-KAT5 antibody	abcam	ab23886
Anti-NLRP3 antibody	Adipogen	Cryo-2
Anti-NLRP3 antibody	CST	15101
Anti-ASC antibody	Adipogen	AL177
Anti-β-actin antibody	Cell Signaling Technology	4967
Anti-NEK7 antibody	abcam	ab133514
Anti-GOLGA4 antibody	Abclonal	A10216
Anti-TGN38 antibody	NOVUS	NBP1-03495
Anti-acetyl lysine mouse mAb(clone Kac-01) antibody	PTM BIO	PTM-101
Anti-DDDDK-tag	MBL	M185-3L
Anti-Myc-tag	MBL	M047-3
Mouse anti GST-Tag mAb	ABclonal	AE001
Cy3–conjugated Affinipure Goat Anti-Rabbit IgG(H+L)	Protein-tech	SA00009-2
Alexa Fluor® 488 Goat anti-mouse IgG (minimal x-reactivity) Antibody	Biolegend	405319
Alexa Fluor 594-conjugated secondary antibody	Biolegend	405326
DAPI	Beyotime	P0131
PE anti-mouse Ly-6C Antibody	Biolegend	128007
FITC anti-mouse/human CD11b Antibody	Biolegend	101205
Chemicals, Peptides, and Recombinant Proteins		
Ultrapure LPS (E. coli 0111:B4)	InvivoGen	tlrl-3pelps
Lipopolysaccharide derived from Escherichia coli 0111:B4	Sigma	L2630
ATP	InvivoGen	tlrl-atpl
nigericin	InvivoGen	tlrl-nig
MSU	InvivoGen	tlrl-msu
FLA-ST	InvivoGen	tlrl-stfla
poly(dA:dT) naked	InvivoGen	tlrl-patn
Imiquimod	Invivogen	R837
nicotinamide	Selleck	S1899
trichostatin A	Selleck	S1045
MB-3	MCE	HY-129039
WM-119	Sigma	SML3067
SI-2 hydrochloride	MCE	HY-101447A
PF-CBP1-HCL	Selleck	S8180
C646	Selleck	S7152
CPI-637	Selleck	S8190
Curcumin	Selleck	S1848

SGC-CBP30	Selleck	S7256
Acetyl-CoA	Sigma	A2181
Lipofectamine 3000 Transfection Reagent	ThermoFisher Scientific	L3000015
NU9056	Tocris	4903
Cell Lysis Buffer	Cell Signaling Technology	9803
Mouse immunoglobin IgG protein	Abcam	ab198772
Protein A/G PLUS-Agarose	Santa cruz	sc-2003
Glutathione Sepharose™ 4B	GE Healthcare	17-0756-01
Recombinant murine NLRP3-His protein	Sino Biological Inc	N/A
Recombinant murine KAT5-GST protein	Sino Biological Inc	N/A
Recombinant GST protein	Sino Biological Inc	N/A
Pierce™ Anti-c-Myc Agarose	ThermoFisher Scientific	20168
Anti-Flag affinity gel	Sigma	A2220
pLenti-CRISPR v2	Addgene	#52961
Lipofectamine™ RNAiMAX	ThermoFisher Scientific	13778030
First-Strand cDNA Synthesis SuperMix	TransGen Biotech	AT34
SYBR qPCR Master Mix	Vazyme Biotech	Q711-02/03
**Critical Commercial Assays**		
Mouse IL-1β ELISA kit	eBioscience	88-7013
Mouse TNF-α ELISA kit	eBioscience	88-7324
BeyoGel™ Blue Native Precast PAGE Gel	Beyotime	P0545S
LDH Cytotoxicity Assay Kit	Beyotime	C0017
**Experimental Models: Cell Lines**		
Mouse Macrophages	Prepared in B.L. Lab	Described in current manuscript
HEK293T cells	American Type Culture Collection (Manassas, VA)	N/A
COS-7 cells	American Type Culture Collection (Manassas, VA)	N/A
NLRP3$^{-/-}$ iBMDM cells	Prepared in B.L. Lab	Described in current manuscript
**Experimental Models: Organisms/Strains**		
C57BL/6 mice	Hunan SJA Laboratory Animal Co.Ltd	N/A
KAT5$^{fl/fl}$	Prepared in Deepak Bararia Lab	Described in current manuscript
KAT5$^{S86A/S86A}$	Prepared in Shengcai Li Lab	Described in current manuscript
Lyz2-Cre	Jackson laboratories	N/A
**Oligonucleotides**		
CCACACUGCAGUAUCUCAATT	Sangon Biotech Co.	KAT5-specific siRNA
UUCUCCGAACGUGUCACGUTT	Sangon Biotech Co.	Control siRNA
5’-CTGCAACGCCACTTGACCAAA-3’	Genechem Co	KAT5-specific shRNA1
5’- CTGCTTATTGAGTTCAGCTAT -3’	Genechem Co	KAT5-specific shRNA2
5’-TTCTCCGAACGTGTCACGT-3’	Genechem Co	Control shRNA
Fwd：5’-TCCCGGTCCAGATCACACTC-3’	Sangon Biotech Co.	KAT5-specific primers

Rev：5’-ACCTTCCGTTTCGTTGAGCG-3’		
Fwd：5’-CTTGTCAGGGGATGAACTCAAAATT-3’<br>Rev：5’-GCCATACGACTCCAG ATAGTAGC-3’	Sangon Biotech Co.	ASC-specific primers
Fwd：5’-ACAAGGCACGGG ACCTATG-3’<br>Rev：5’-TCCCAGTCAGTCCTGGAAATG-3’	Sangon Biotech Co.	caspase-1-specific primers
Fwd：5’-TGGATGGGTTTGCTGGGAT-3’<br>Rev：5’-CTGCGTGTAGCGACTGTTGAG-3’	Sangon Biotech Co.	NLRP3-specific primers
Fwd：5’- GCAACTGTTCCTGAACTCAACT-3’<br>Rev：5’- ATCTTTTGGGGTCCGTCAACT-3’	Sangon Biotech Co.	IL-1β-specific primers
Fwd：5’-AGTGTGACGTTGACATCCGT-3’<br>Rev：5’-GCAGCTCAGTAACAGTCCGC-3’	Sangon Biotech Co.	β-actin-specific primers
Fwd：5’-GACCTCAAGAAATTCAGAATGCATTTGGAAGAT-3<br>Rev：5’-ATCTTCCAAATGCATTCTGAATTTCTTGAGGTC-3’	Sangon Biotech Co.	NLRP3-K24R specific primers
Fwd：5’-ACCATCCTAGCCAGGAGGATTATGTTGGACTGG-3’<br>Rev：5’-CCAGTCCAACATAATCCTCCTGGCTAGGATGGT-3’	Sangon Biotech Co.	NLRP3-K234R specific primers
Fwd：5’-CAAGTTTTGTGTGAAAGGATGAAGGACCCACAG-3’<br>Rev：5’-CTGTGGGTCCTTCATCCTTTCACACAAAACTTG-3’	Sangon Biotech Co.	NLRP3-K875R specific primers
**Software and Algorithms**		
Graphpad Prism 9 software	Graphpad Prism 8 software	N/A
Adobe Illustrator CS6	Adobe	N/A
Adobe Photoshop CC	Adobe	N/A
Microsoft Excel	Microsoft	N/A
