# Supplementary material for: Acetylation is required for full activation of the NLRP3 inflammasome
Source: Nat Commun. 2023 Dec 18;14:8396. doi: 10.1038/s41467-023-44203-0 (PMC10728138; doi:10.1038/s41467-023-44203-0)
Supplement: Supplementary file 3 — Description of Additional Supplementary Files [file 41467_2023_44203_MOESM3_ESM.pdf]

Description of Additional Supplementary Files

File Name: Supplementary Movie 1
Description: **The Live cell imaging of COS-7 Cells transfected with GFP-vector under nigericin stimulation**
COS-7 Cells were transfected with Vector-GFP plasmid, 36 hours later, the cells were stimulated with nigericin (10 µM). Images were taken every 5 min and last Validate for 2 h under ZEISS Axio observer7 microscopy. Image size, 150 µm x 140 µm.

File Name: Supplementary Movie 2
Description: **The Live cell imaging of COS-7 Cells transfected with NLRP3 WT-GFP-vector under nigericin stimulation**
COS-7 Cells were transfected with NLRP3-WT-GFP plasmid, 36 hours later, the cells were stimulated with nigericin (10 µM). Images were taken every 5 min and last Validate for 2 h under ZEISS Axio observer7 microscopy. Image size, 150 µm x 140 µm.

File Name: Supplementary Movie 3
Description: **The Live cell imaging of COS-7 Cells transfected with NLRP3 K24R-GFP-vector under nigericin stimulation**
COS-7 Cells were transfected with NLRP3-K24R-GFP plasmid, 36 hours later, the cells were stimulated with nigericin (10 µM). Images were taken every 5 min and last Validate for 2 h under ZEISS Axio observer7 microscopy. Image size, 150 µm x 140 µm.
