# Supplementary material for: Acetylation is required for full activation of the NLRP3 inflammasome
Source: Nat Commun. 2023 Dec 18;14:8396. doi: 10.1038/s41467-023-44203-0 (PMC10728138; doi:10.1038/s41467-023-44203-0)
Supplement: Supplementary file 7 — Reporting Summary [file 41467_2023_44203_MOESM7_ESM.pdf]

nature portfolio

Corresponding author(s): Kai Zhao

Last updated by author(s): 11/19/2023

Reporting Summary

Nature Portfolio wishes to improve the reproducibility of the work that we publish. This form provides structure for consistency and transparency in reporting. For further information on Nature Portfolio policies, see our Editorial Policies and the Editorial Policy Checklist.

Statistics

For all statistical analyses, confirm that the following items are present in the figure legend, table legend, main text, or Methods section.

n/a	Confirmed	
☐	☒	The exact sample size (*n*) for each experimental group/condition, given as a discrete number and unit of measurement
☐	☒	A statement on whether measurements were taken from distinct samples or whether the same sample was measured repeatedly
☐	☒	The statistical test(s) used AND whether they are one- or two-sided<br>*Only common tests should be described solely by name; describe more complex techniques in the Methods section.*
☒	☐	A description of all covariates tested
☐	☒	A description of any assumptions or corrections, such as tests of normality and adjustment for multiple comparisons
☐	☒	A full description of the statistical parameters including central tendency (e.g. means) or other basic estimates (e.g. regression coefficient) AND variation (e.g. standard deviation) or associated estimates of uncertainty (e.g. confidence intervals)
☐	☒	For null hypothesis testing, the test statistic (e.g. *F*, *t*, *r*) with confidence intervals, effect sizes, degrees of freedom and *P* value noted<br>*Give P values as exact values whenever suitable.*
☒	☐	For Bayesian analysis, information on the choice of priors and Markov chain Monte Carlo settings
☒	☐	For hierarchical and complex designs, identification of the appropriate level for tests and full reporting of outcomes
☒	☐	Estimates of effect sizes (e.g. Cohen's *d*, Pearson's *r*), indicating how they were calculated

Our web collection on statistics for biologists contains articles on many of the points above.

Software and code

Policy information about availability of computer code

Data collection	ELISA and data of LDH release were collected using TECAN Spark®. Flow cytometry data were collected using BD LSRFORTESSA. Western Blot data were collected using ChemiDoc Imaging System. Mass spectrometry data were collected using Q ExactiveTM Plus mass spectrometry (Thermo) coupled to an ekspert EASY-nLC 1000 (Thermo).Immunofluorescence of protein colocalization data were collected using (SpinSR10; Olympus) and (LSM800; ZEISS). Living cell imaging data were collected using ZEISS Axio observer7.
Data analysis	Mass spectrometry data were processed using Proteome Discoverer 1.3. Statistical analaysis were performed with GraphPad Prism 9.4.1. Flow cytometry data analysis was performed using Flowjo v10. The immunofluorescence images were statistically analyzed by Zen 3.6 and Fiji image J (Version 2.9.0)

For manuscripts utilizing custom algorithms or software that are central to the research but not yet described in published literature, software must be made available to editors and reviewers. We strongly encourage code deposition in a community repository (e.g. GitHub). See the Nature Portfolio guidelines for submitting code & software for further information.

Data

Policy information about availability of data

All manuscripts must include a data availability statement. This statement should provide the following information, where applicable:

- Accession codes, unique identifiers, or web links for publicly available datasets
- A description of any restrictions on data availability
- For clinical datasets or third party data, please ensure that the statement adheres to our policy

All data supporting the findings of this study are available within the paper and its Supplementary Information;The mass spectrometry data have been deposited to the ProteomeXchange Consortium via the PRIDE partner repository with the accession code PXD041763.

Research involving human participants, their data, or biological material

Policy information about studies with human participants or human data. See also policy information about sex, gender (identity/presentation), and sexual orientation and race, ethnicity and racism.

Reporting on sex and gender	Not applicable
Reporting on race, ethnicity, or other socially relevant groupings	Not applicable
Population characteristics	Not applicable
Recruitment	Not applicable
Ethics oversight	Not applicable

Note that full information on the approval of the study protocol must also be provided in the manuscript.

Field-specific reporting

Please select the one below that is the best fit for your research. If you are not sure, read the appropriate sections before making your selection.

☒ Life sciences ☐ Behavioural & social sciences ☐ Ecological, evolutionary & environmental sciences

For a reference copy of the document with all sections, see nature.com/documents/nr-reporting-summary-flat.pdf

Life sciences study design

All studies must disclose on these points even when the disclosure is negative.

Sample size	Sample size for each experiment is indicated in the legend. In general, no calculations were done to determine sample size. Sample size of cellular experiments was determined based on standards for experimental cell biology, attempting to have a minimum of N = 3 biological replicates with sufficient reproducibility. For animal studies, the size of sample was determined by available age- and sex matched mice with genotype difference. Statistical significance is reported in the manuscript.
Data exclusions	No data exclusions.
Replication	All experimental findings were replicated at least 3 times with enough reproducibility. All attempts at data replication were successful.
Randomization	For in vitro experiments, mouse macrophages were isolated from randomly chosen wild-type mice, genetically engineered mouse including Kat5S86A/S86A, Kat5fl/fllyz2-Cre, Nlrp3K24R/K24R mice and their corresponding WT littermates' mice, processing was performed simultaneously and in parallel for all conditions within each experiment, equal cells were allocated randomly for culture and analysis. All animals used in this manuscript were allocated into experimental groups randomly.
Blinding	A researcher blinded to the group allocation was responsible for the data collection and final data analysis.

Reporting for specific materials, systems and methods

We require information from authors about some types of materials, experimental systems and methods used in many studies. Here, indicate whether each material, system or method listed is relevant to your study. If you are not sure if a list item applies to your research, read the appropriate section before selecting a response.

Materials & experimental systems

n/a	Involved in the study
☐	☒ Antibodies
☐	☒ Eukaryotic cell lines
☒	☐ Palaeontology and archaeology
☐	☒ Animals and other organisms
☒	☐ Clinical data
☒	☐ Dual use research of concern
☒	☐ Plants

Methods

n/a	Involved in the study
☒	☐ ChIP-seq
☐	☒ Flow cytometry
☒	☐ MRI-based neuroimaging

Antibodies

Antibodies used

Anti-caspase-1(1:1000 for WB, # ab179515), Anti-KAT5(1:1000 for WB,1:200 for immunofluorescences # ab23886), Anti-NEK7 (1:1000 for WB, # ab133514) were purchased from Abcam. Anti-NLRP3(1:1000 for WB, 1:200 for immunofluorescences # Cryo-2) Anti-ASC(1:1000 for WB, # AL177) were purchased from Adipogen. Anti-β-actin antibody(1:10000 for WB, # B4967), Anti-NLRP3(1:1000 for WB, #15101) were purchased from Cell Signaling Technology.Anti-IL-1β (1:1000 for WB, # AF-401-NA;) was purchased from RD systems Anti-GOLGA4(1:200 for immunofluorescences, # A10216), Mouse anti GST-Tag(1:200 for WB, # AE001) were purchased from Abclonal.Anti-TGN38(1:200 for immunofluorescences, # NBP1-03495) were purchased from NOVUS.Anti-acetyl lysine mouse mAb(clone Kac-01)(1:500 for WB, #PTM-101) was purchased from PTM BIO. Anti-DDDDK(1:1000 for WB, # M185-3L), Anti-Myc(1:1000 for WB, #M047-3) were purchased from MBL. Cy3–conjugated Affinipure Goat Anti-Rabbit IgG(H+L)(1:100 for immunofluorescences, #SA00009-2) was purchased from Protein-tech. Alexa Fluor® 488 Goat anti-mouse IgG (minimal x-reactivity) Antibody(1:50 for immunofluorescences, #405319), Alexa Fluor 594-conjugated secondary antibody (1:50 for immunofluorescences, #405326) ,PE anti-mouse Ly-6C Antibody(1:500 for flowcytometry, #128007), FITC anti-mouse/human CD11b(1:1000 for flowcytometry, #101205) were purchased from Biolegend. DAPI(1:1 for immunofluorescences, # P0131) was purchased from Beyotime.
The antibody of Acetyl-NLRP3-K24 was customized produced by ABclonal (Wuhan, China). It was generated by immunizing rabbits with the acetyl-lysine-peptide KF(K-Ac)-Nle-HLED-C, covalently cross-linked to keyhole limpet hemocyanin (KLH).

Validation

Anti-caspase-1(1:1000 Validate for WB, # ab179515), https://www.abcam.cn/products/primary-antibodies/pro-caspase-1--p10--p12-antibody-epr16883-ab179515.html
Anti-KAT5(1:1000 Validate for WB,1:200 Validate for immunofluorescences # ab23886) https://www.abcam.cn/products/primary-antibodies/kat5--tip60-antibody-ab23886.html
Anti-NEK7 (1:1000 Validate for WB, # ab133514) https://www.abcam.cn/products/primary-antibodies/nek7-antibody-epr4900-ab133514.html
Anti-NLRP3(1:1000 Validate for WB, 1:200 Validate for immunofluorescences # Cryo-2) https://adipogen.com/ag-20b-0014-anti-nlrp3-nalp3-mab-cryo-2.html
Anti-ASC(1:1000 Validate for WB, # AL177) https://adipogen.com/ag-25b-0006-anti-asc-pab-al177.html
Anti-β-actin antibody(1:10000 Validate for WB, #4967) https://www.cellsignal.cn/products/primary-antibodies/b-actin-antibody/4967
Anti-NLRP3(1:1000 Validate for WB, #15101) https://www.cellsignal.cn/products/primary-antibodies/nlrp3-d4d8t-rabbit-mab/15101
Anti-IL-1β (1:1000 Validate for WB, # AF-401-NA) https://www.rndsystems.com/cn/products/mouse-il-1beta-il-1f2-antibody_af-401-na
Anti-GOLGA4(1:200 Validate for immunofluorescences, # A10216) https://abclonal.com.cn/catalog/A10216
Mouse anti GST-Tag(1:200 Validate for WB, # AE001) https://abclonal.com.cn/catalog/AE001.
Anti-TGN38(1:200 Validate for immunofluorescences, #NBP1-03495) https://www.novusbio.com/products/tgn38-antibody_nbp1-03495.
Anti-acetyl lysine mouse mAb(clone Kac-01)(1:500 Validate for WB, #PTM-101) http://www.ptm-biolab.com.cn/productDetail.html?id=4610.
Anti-DDDDK (1:1000 Validate for WB, # M185-3L) https://www.mbl-chinawide.cn/search012?keyword=M185-3L
Anti-Myc(1:1000 Validate for WB, #M047-3) https://www.mbl-chinawide.cn/search012?keyword=M047-3
Cy3–conjugated Affinipure Goat Anti-Rabbit IgG(H+L)(1:100 Validate for immunofluorescences, #SA00009-2) https://www.ptgcn.com/products/Cy3-conjugated-Affinipure-Goat-Anti-Rabbit-IgG-H-L-secondary-antibody.htm
Alexa Fluor® 488 Goat anti-mouse IgG (minimal x-reactivity) Antibody(1:50 Validate for immunofluorescences, #405319) https://www.biolegend.com/en-us/products/alexa-fluor-488-goat-anti-mouse-igg-minimal-x-reactivity-9282
Alexa Fluor 594-conjugated secondary antibody (1:50 Validate for immunofluorescences, #405326) https://www.biolegend.com/en-us/products/alexa-fluor-594-goat-anti-mouse-igg-minimal-x-reactivity-9706
DAPI (1:1 Validate for immunofluorescences, # P0131) https://www.beyotime.com/product/P0131-5ml.htm
PE anti-mouse Ly-6C Antibody (1:500 Validate for flowcytometry, #128007) https://www.biolegend.com/en-us/products/pe-anti-mouse-ly-6c-antibody-4904
FITC anti-mouse/human CD11b(1:1000 Validate for flowcytometry, #101205) https://www.biolegend.com/en-us/products/fitc-anti-mouse-human-cd11b-antibody-347

The Acetyl-NLRP3-K24 (1:200 dilution) was successfully validated for the detection of the K24 acety-NLRP3 peptides and over-expression experiments in 293T cells in the manuscript(Figure S1d,e).

Eukaryotic cell lines

Policy information about cell lines and Sex and Gender in Research

Cell line source(s)	HEK293T cells and COS-7 cells were obtained from ATCC; iBMDMs were provided by Dr. Feng Shao.
Authentication	All cell lines were authenticated by STR profiling .
Mycoplasma contamination	All cell lines were routinely verified to be free of mycoplasma contamination
Commonly misidentified lines (See ICLAC register)	No commonly misidentified cell lines were used in this study.

Animals and other research organisms

Policy information about studies involving animals; ARRIVE guidelines recommended for reporting animal research, and Sex and Gender in Research

Laboratory animals	Wild-type C57BL/6J mice 6-8 weeks old were purchased from Hunan SJA Laboratory Animal Co. Ltd (Changsha, China) (transferred from National Rodent Laboratory Animal Resources center).Lyz2-Cre mice(Jackson Labs, stock no. 004781)were purchased from Jackson laboratories. Kat5fl/fl and Kat5S86A/S86A mice were gifts from Professor Deepak Bararia(Blood 136, 1735-1747 (2020).) and Professor Shengcai Lin(Nature communications 9, 1916 (2018).) All mice with same gender were used between 6 and 8 weeks of age. They were housed in a 12-h dark/light cycle (25 ± 2℃) under specific pathogen-free conditions with unrestricted access to food and water.The experimental and control animals were co-housed.
Wild animals	The study did not involve wild animals.
Reporting on sex	Female mice were used for experiments.
Field-collected samples	The study did not involve samples collected from the field.
Ethics oversight	Animal experiments were conducted in accordance with the Institutional Animal Care and Use Committee of Central South University (NO.2018sydw0344)

Note that full information on the approval of the study protocol must also be provided in the manuscript.

Flow Cytometry

Plots

Confirm that:

☒ The axis labels state the marker and fluorochrome used (e.g. CD4-FITC).

☒ The axis scales are clearly visible. Include numbers along axes only for bottom left plot of group (a 'group' is an analysis of identical markers).

☒ All plots are contour plots with outliers or pseudocolor plots.

☒ A numerical value for number of cells or percentage (with statistics) is provided.

Methodology

Sample preparation	After counting the number of harvested peritoneal exudate cells, the cells were washed with PBS, then resuspended with 100 µl binding buffer (1× PBS, 2% FBS) softly; FITC anti-mouse/human CD11b Antibody (Biolegend 101205)and PE anti-mouse Ly-6C Antibody(Biolegend 128007) were added and incubated in a dark room for 30min.After washing three times with PBS, stained cells were analyzed on the BD LSRFortessa to detect the numbers of neutrophils.
Instrument	BD LSRFortessa
Software	FlowJo v10
Cell population abundance	Neutrophils are FITC/PE-double-positive.
Gating strategy	Live cells were select by FSC and SSC, and then analyzed by FITC and PE. Neutrophils are FITC/PE-double-positive.

☒ Tick this box to confirm that a figure exemplifying the gating strategy is provided in the Supplementary Information.
